# BMP2 gene transfer induces pericardial effusion and inflammatory response in the ischemic porcine myocardium

**DOI:** 10.3389/fcvm.2023.1279613

**Published:** 2023-11-03

**Authors:** H. H. Pulkkinen, A. Kivistö-Rahnasto, H. Korpela, M. Heikkilä, N. Järveläinen, S. Siimes, L. Kilpeläinen, N. Laham-Karam, S. Ylä-Herttuala, J. P. Laakkonen

**Affiliations:** ^1^ A.I. Virtanen Institute for Molecular Sciences, University of Eastern Finland, Kuopio, Finland; ^2^ Heart Center, Kuopio University Hospital, Kuopio, Finland; ^3^ Gene Therapy Unit, Kuopio University Hospital, Kuopio, Finland

**Keywords:** bone morphogenetic protein 2, BMP2, myocardial ischemia, ischemic heart disease, gene therapy, inflammation, pig myocardium, coronary artery disease

## Abstract

Pro-angiogenic gene therapy is being developed to treat coronary artery disease (CAD). We recently showed that bone morphogenetic protein 2 (BMP2) and vascular endothelial growth factor-A synergistically regulate endothelial cell sprouting *in vitro*. BMP2 was also shown to induce endocardial angiogenesis in neonatal mice post-myocardial infarction. In this study, we investigated the potential of BMP2 gene transfer to improve cardiomyocyte function and neovessel formation in a pig chronic myocardial infarction model. Ischemia was induced in domestic pigs by placing a bottleneck stent in the proximal part of the left anterior descending artery 14 days before gene transfer. Intramyocardial gene transfers with adenovirus vectors (1 × 10^12^ viral particles/pig) containing either human BMP2 (AdBMP2) or beta-galactosidase (AdLacZ) control gene were performed using a needle injection catheter. BMP2 transgene expression in the myocardium was detected with immunofluorescence staining in the gene transfer area 6 days after AdBMP2 administration. BMP2 gene transfer did not induce angiogenesis or cardiomyocyte proliferation in the ischemic pig myocardium as determined by the quantitations of CD31 or Ki-67 stainings, respectively. Accordingly, no changes in heart contractility were detected in left ventricular ejection fraction and strain measurements. However, BMP2 gene transfer induced pericardial effusion (AdBMP2: 9.41 ± 3.17 mm; AdLacZ: 3.07 ± 1.33 mm) that was measured by echocardiography. Furthermore, an increase in the number of immune cells and CD3^+^ T cells was found in the BMP2 gene transfer area. No changes were detected in the clinical chemistry analysis of pig serum or histology of the major organs, implicating that the gene transfer did not induce general toxicity, myocardial injury, or off-target effects. Finally, the levels of fibrosis and cardiomyocyte apoptosis detected by Sirius red or caspase 3 stainings, respectively, remained unaltered between the groups. Our results demonstrate that BMP2 gene transfer causes inflammatory changes and pericardial effusion in the adult ischemic myocardium, which thus does not support its therapeutic use in chronic CAD.

## 1. Introduction

In coronary artery disease (CAD), the blood flow in the coronary arteries is reduced because of atherosclerotic plaques, which can eventually lead to myocardial ischemia and myocardial infarction (MI), the most serious complication of CAD. The first-line treatment for CAD is medication using nitrates, which induces vessel dilatation. Blocked arteries can also be treated with percutaneous coronary intervention, a procedure that opens narrowed or blocked vessels, or coronary artery bypass grafting, in which a healthy vessel from another location in the body is used to bypass the blocked artery. Since conventional therapies are not suitable for every patient with severe myocardial ischemia, other therapeutic strategies that focus on increasing angiogenesis, improving the recovery of cardiomyocytes (CMs), or reducing inflammation of the ischemic heart have been developed for CAD (1–5).

Previously, catheter-mediated gene transfer (GT) into the heart using vascular endothelial growth factors (VEGFs) was successfully used to induce angiogenesis in the pig myocardium (6–8). Particularly, gene therapy using VEGF-D has shown promise in phase I/IIa clinical trials in refractory angina patients by increasing myocardial perfusion (9, 10). Other angiogenic or regenerative growth factors, such as fibroblast growth factors (FGFs), hepatocyte growth factors, and stromal cell-derived factor 1 (CXCL12), have also been used to induce therapeutic myocardial vascular growth or improve CM function (11–13) (NCT03404024). Aside from positive treatment outcomes, adverse side effects, such as edema or arrhythmia, were observed after intramyocardial GT with specific growth factors in preclinical studies (14, 15). Most of the used transgenes have also failed to show beneficial therapeutic effects in clinical trials, or the results have been ambiguous despite promising outcomes in preclinical studies (3). Thus, treating CAD requires novel transgenes.

Bone morphogenetic proteins (BMPs) are a group of growth factors known to regulate osteogenesis (16, 17), heart development (18), and angiogenesis (19–24). They bind to BMP receptor I (BMPRI) and II (BMPRII) and initiate the SMAD, PI3K, and MAPK signaling cascades (25). Bone morphogenetic growth factor 2 (BMP2) has been widely studied in the context of osteogenesis, and the growth factor has been clinically approved as a treatment for bone regeneration (26). Aside from being an osteogenic factor, BMP2 has been shown to be a pro-angiogenic factor in several *in vitro* and *in vivo* settings (21, 23, 24, 27–29). Furthermore, we recently demonstrated that BMP2 regulates VEGFR2 signaling in endothelial cells and that BMP2 is upregulated after vascular endothelial growth factor-A (VEGF-A) GT in mice (21). In addition, BMP2 has been shown to induce CM proliferation, differentiation, and functionality (30–32). Due to the increasing evidence of the regulatory roles of BMP2 in angiogenesis and CM functionality, interest in utilizing BMP2 as a treatment for CAD and the underlying myocardial ischemia has emerged (20, 32, 33). For example, in an acute myocardial ischemia model of an injured neonatal mouse, BMP2 induced endocardial angiogenesis (20). In another study, systemic delivery of recombinant BMP2 protein shortly after MI limited CM apoptosis without affecting the heart functionality in an acute myocardial ischemia model in adult mice (32). To date, the potential therapeutic effect of the BMP2 transgene has not been studied in adult large animal models of acute or chronic myocardial ischemia.

Based on the data described above and our earlier findings of the synergistic effects of VEGF-A and BMP2 (21), we designed an adenovirus vector that expresses human BMP2 (hBMP2) transgene. We studied its potential for gene therapy to treat chronic myocardial ischemia in a large animal model. We hypothesized that local, intramyocardial BMP2 GT could induce angiogenesis, limit CM apoptosis, and improve the functionality of the adult ischemic pig myocardium.

## 2. Results

### 2.1. BMP2 transgene is successfully expressed in the ischemic pig myocardium after GT

Adenovirus vectors with human BMP2 (AdBMP2) transgene under a cytomegalovirus (CMV) promoter were generated and produced according to the standard methods in the EATRIS-ERIC infrastructure, National Virus Vector Laboratory (NVVL, Kuopio, Finland). Prior to animal experiments, Western blot (WB) and ELISA confirmed the expression and secretion of the human BMP2 protein from AdBMP2-transduced primary endothelial cells (HUVECs) (Supplementary Figure S1).

**Figure 1:**
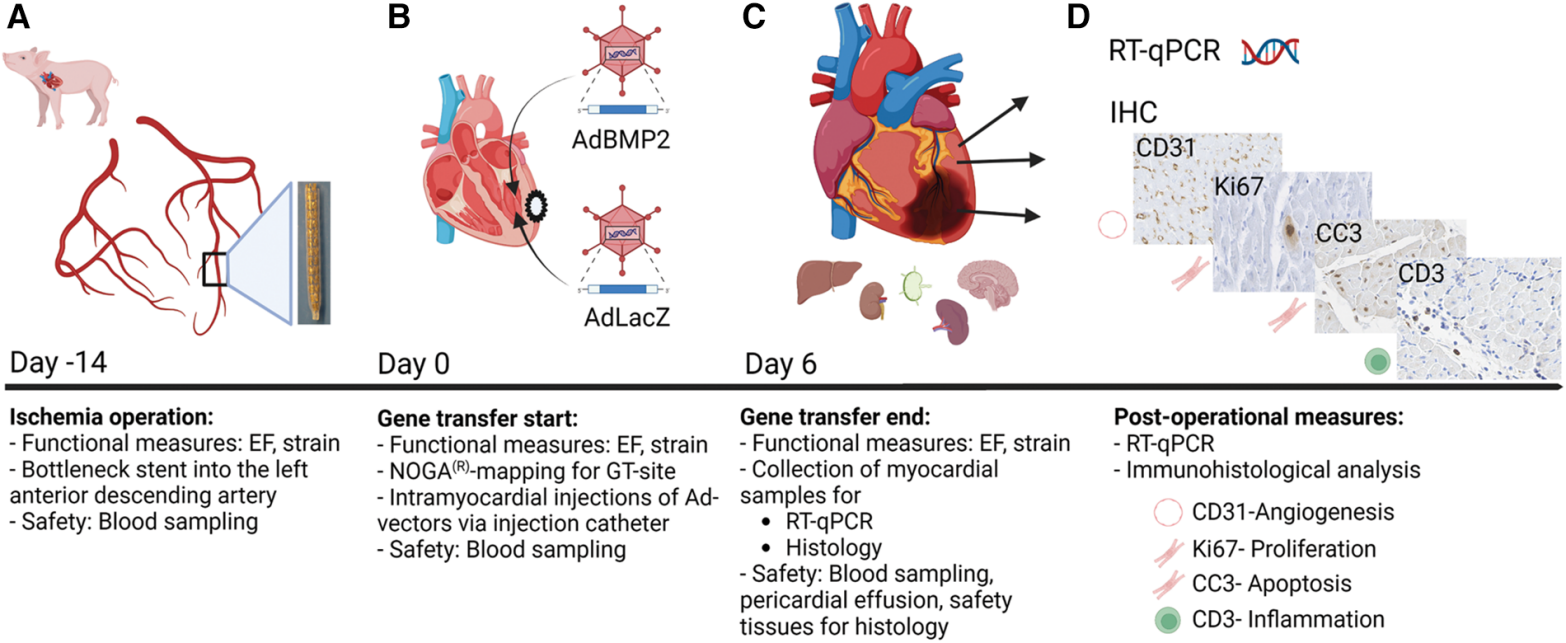
Study protocol for BMP2 gene transfer in ischemic pig myocardium. Functional measurements and blood sampling were done at all time points: on day −14 (ischemia operation), day 0 (gene transfer start), and day 6 (gene transfer end). Left ventricular ejection fraction (EF) and strain measurements were taken to evaluate the heart functionality. (**A**) Ischemia operation was performed 2 weeks before the gene transfer (day −14). A bottleneck stent was introduced to the left anterior descending artery (LAD) of the porcine hearts to limit perfusion and create myocardial ischemia. (**B**) Gene transfer started on day 0. The optimal gene transfer site (GT site) was detected using the NOGA mapping system. Injection catheters were used to inject AdBMP2 or AdLacZ vectors into the ischemic myocardium. (**C,D**) Six days after the gene transfer (day 6), the pigs were sacrificed, and samples were collected for post-operational measures. Myocardial samples were collected for RT-qPCR and histological analysis. Safety tissues were collected for histological analysis to determine the safety of the gene transfer. The size of pericardial effusion was measured to evaluate the safety of the GT on day 6. Image created with BioRender.com.

Ischemia in the pig myocardium was induced by placing a bottleneck stent in the proximal part of the left anterior descending artery 14 days before GT, as our previous study described (34) (Figure 1A). An electroanatomical map of the left ventricle was acquired using the NOGA® mapping system to detect the ischemic area in each animal prior to GT (Figure 1B). Adenovirus vectors [AdBMP2 or AdLacZ control, 1 × 10^12^ viral particles (vp)/pig] were administrated to the left ventricle using an intramyocardial injection catheter. Left ventricular ejection fraction and strain analysis were measured before ischemia operation, during GT, and immediately prior to sacrifice (Figures 1A–C). Tissue samples from the heart were collected from the GT area, GT edge, and posterior wall for immunohistological and RT-qPCR analyses (Figures 1C,D, *n* = 5 pigs/group). Blood samples were collected before the ischemia operation (d−14), GT (d0), and sacrifice (d6, Figures 1A–C). Tissue samples from the lung, liver, kidney, spleen, brain, retina, ovary, and lymph nodes were collected to analyze the safety of GT (Figure 1C).

**Figure 2:**
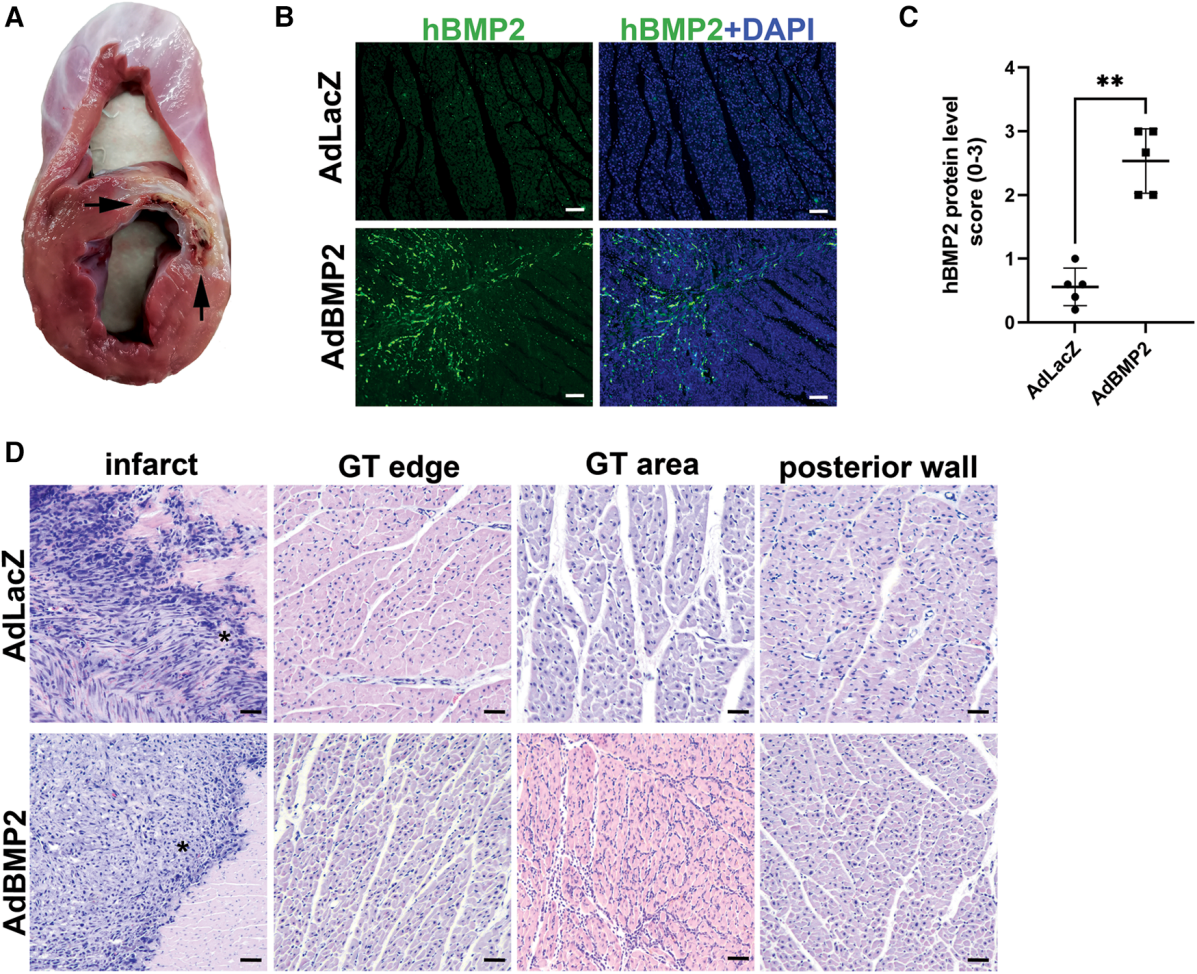
BMP2 transgene is successfully expressed in the pig myocardium after gene transfer. (**A**) Bottleneck stent in LAD caused ischemia and infarct of the anterior wall of the left ventricle and interventricular septum. Infarcted areas are marked with arrows. (**B**) Representative images of immunofluorescence staining of hBMP2 protein expression (BMP2 Ab, green) in the ischemic myocardium of AdLacZ- and AdBMP2-treated pigs at d6 after gene transfer (GT). Nuclei are stained with DAPI (blue). Scale bars, 100 µm. (**C**) Scoring of hBMP2 protein expression in AdLacZ- and AdBMP2-treated ischemic pig myocardium (five pigs/group, AdBMP2 = 16 samples, AdLacZ = 23 samples). hBMP2 protein was expressed in AdBMP2 (average score 2.53) but not in AdLacZ (average score 0.56)-treated hearts. Scores 0–3 were used, where 0 = absent, 1 = low, 2 = moderate, and 3 = high hBMP2 expression. The mean ± SD values are shown. The Mann–Whitney *U* test was used to determine the statistical significance. **p < 0.01. (**D**) Representative H&E images of the ischemic myocardium of AdLacZ- and AdBMP2-treated pigs at d6 after GT. Images from the infract area (*), border of the GT area (GT edge), GT area, and left ventricle posterior wall (negative control of GT) and ischemia are shown. Scale bars, 100 µm.

The bottleneck stent in the LAD in the pig myocardium generated ischemia within 20 days, as indicated in Figure 2A. The ischemia or GT with the AdBMP2 or AdLacZ groups did not induce mortality. No cardiac arrhythmias were detected with ECG monitoring after the GT operation or during d6 measurements. At d6 after GT, hBMP2 protein expression was detected from immunofluorescence (IF)-stained myocardial samples. hBMP2 protein was expressed in AdBMP2 but not in AdLacZ-treated myocardium (Figures 2B,C). BMP2 protein expression was detected in cells morphologically resembling endothelial cells, fibroblasts, and CMs. After performing H&E staining, the infarction zone was clearly visible, and more inflammatory cells were seen in AdBMP2 GT-treated animals than in the AdLacZ control group (Figure 2D). Endogenous pig BMP2 gene expression was confirmed unaltered because of ischemia and between the AdLacZ and AdBMP2 GT groups by RT-qPCR (Supplementary Figure S2).

### 2.2. BMP2 induces pericardial effusion but does not affect heart functionality

**Figure 3:**
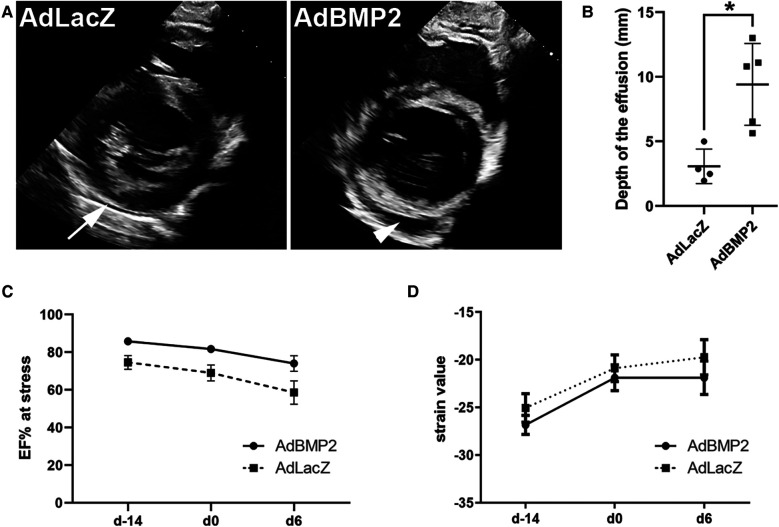
AdBMP2 induces pericardial effusion but has no effect on heart functionality. (**A**) Representative images of pericardial fluid at d6 after AdLacZ and AdBMP2 GT detected by transthoracic echocardiography. Only a physiological amount of pericardial fluid was seen in the AdLacZ group (arrow), whereas AdBMP2-treated animals had visually apparent pericardial fluid accumulation (arrowhead). (**B**) The size of the pericardial fluid effusion was determined in the end-diastole at d6 after GT. The effusion size was clinically relevant in the AdBMP2 group as it was 9.4 ± 3.2 mm. Compared to the AdLacZ group (*n* = 4), the size was significantly larger in the AdBMP2 group (*n* = 5). The mean ± SD values are shown. The Mann–Whitney *U* test was used to determine the statistical significance. *p < 0.05. (**C**) Ejection fraction (EF) under dobutamine-stress was measured at three time points: before ischemia operation (day −14), before GT (day 0), and after the GT (day 6). AdBMP2 GT did not alter EF from day 0 to day 6 (*n* = 3 or 6 pigs/time point). Mean ± SEM values are shown. The Mann–Whitney *U* test was used to determine the statistical significance. (**D**) Heart contractility was determined with strain analysis using the average global strain values from the myocardium taken at three time points: before ischemia operation (day −14), before GT (day 0), and after GT (day 6). Heart contractility did not change due to AdBMP2 (*n* = 5 pigs/time point) GT when compared with the AdLacZ group (*n* = 4–5 pigs/time point). Mean ± SEM values are shown. The Mann–Whitney *U* test was used to determine the statistical significance.

Pathological pericardial fluid accumulation measured by transthoracic echocardiography was visible in all AdBMP2-treated animals at d6 after GT prior to sacrifice, while there were only physiological amounts of pericardial fluid in the AdLacZ control group (Figure 3A). The size of the pericardial effusion in the end-diastole detected by echocardiography was significantly larger in the AdBMP2 group in comparison to the AdLacZ group (9.41 ± 3.17 mm and 3.07 ± 1.33 mm, respectively, *р*-value of 0.008; Figure 3B). No thinning and separation of myocardial fibers were seen in the GT areas (Figure 2D), which could have implied intercellular edema (35).

Heart functionality was measured before the ischemia operation and before and after the GT. No significant changes in the average ejection fractions (EFs) under stress were detected for AdBMP2-treated pigs (81.7% at d0 and 74.0% at d6). For AdLacZ-treated pigs, the values were 69.0% and 58.6%, respectively (Figure 3C and Supplementary Figure S3A). The left ventricle strain values were additionally measured at the levels of the mitral valve, papillary muscles, and apex, which have been suggested to be more sensitive in detecting the contractile function of the heart than traditional M-mode EF measurements (36). The average global strain value was unaltered from d0 to d6 in AdBMP2-treated pigs (−21.9 at day 0 and day 6). For AdLacZ-treated pigs, the global strain values were −20.9 at day 0 and −19.7 at day 6 after GT (Figure 3D and Supplementary Figure S3B). To conclude, AdBMP2 GT did not cause significant changes in the heart functionality but induced pathological pericardial effusion.

### 2.3. BMP2 does not change blood values or induce off-target effects

**Figure 4:**
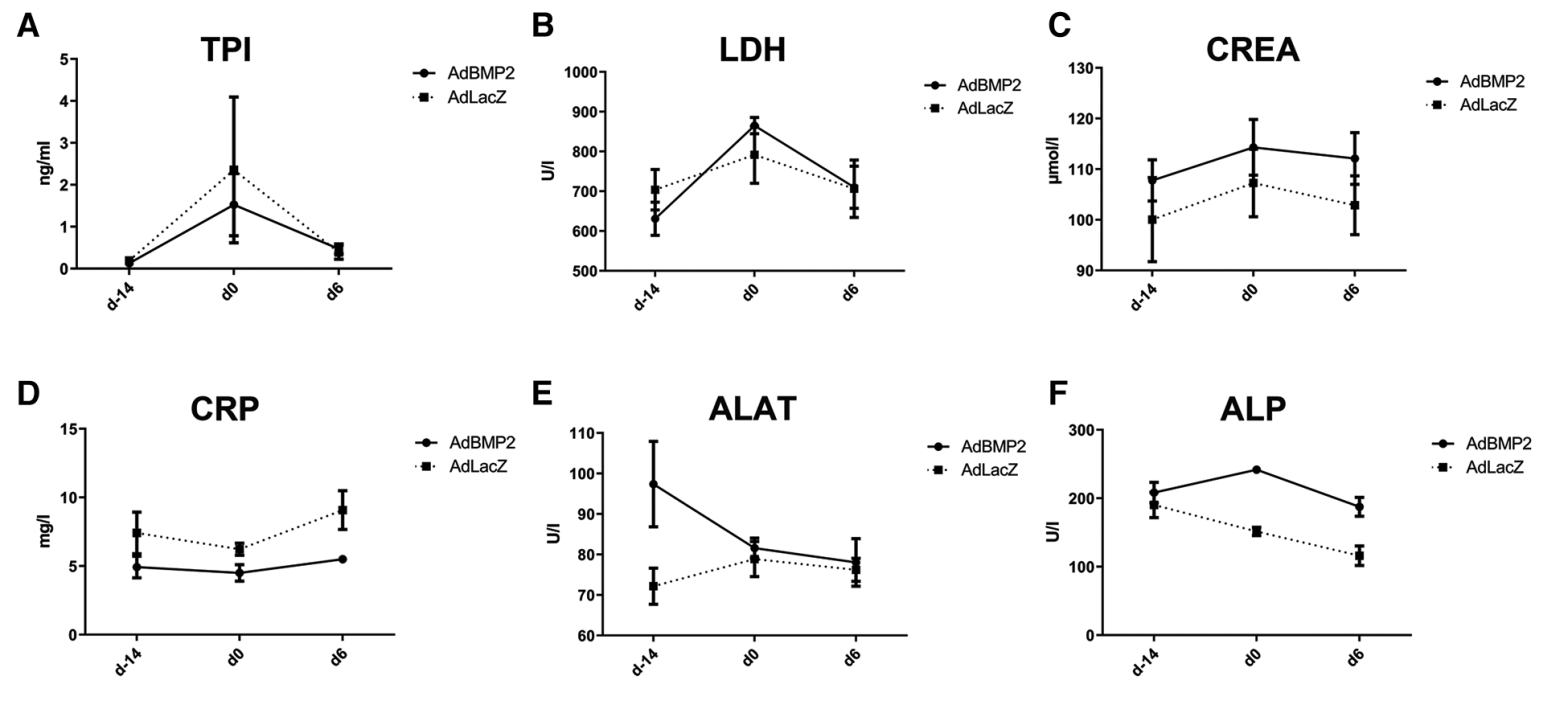
No off-target effects, myocardial injury, or toxicity were detected in AdBMP2-treated pigs. No elevations in (**A**) troponin I (TPI), (**B**) lactate dehydrogenase (LDH), (**C**) creatinine (CREA), (**D**) C-reactive protein (CRP), (**E**) alanine aminotransferase (ALAT), and (**F**) alkaline phosphatase (ALP) were detected. Blood values were measured at three time points: before ischemia operation (day −14), before GT (day 0), and after GT (day 6). Mean ± SEM values for each group are shown (*n* = 4–5 pigs/group).

The safety of AdBMP2 GT on the heart and other organs was further evaluated from blood values from pig sera collected at all three time points. AdBMP2 GT did not increase the serum levels of troponin I (TPI), lactate dehydrogenase (LDH), creatinine (CREA), C-reactive protein (CRP), alanine aminotransferase (ALAT), or alkaline phosphatase (ALP) (Figures 4A–F), indicating that AdBMP2 GT did not induce tissue toxicity or myocardial injury. The histology of the major organs was additionally evaluated to detect possible adverse effects of the GT. No morphological changes in the lung, liver, kidney, spleen, brain, retina, ovary, or lymph node were detected after GT (Supplementary Figure S4).

### 2.4. BMP2 does not induce angiogenesis or affect cardiomyocyte apoptosis or proliferation

**Figure 5:**
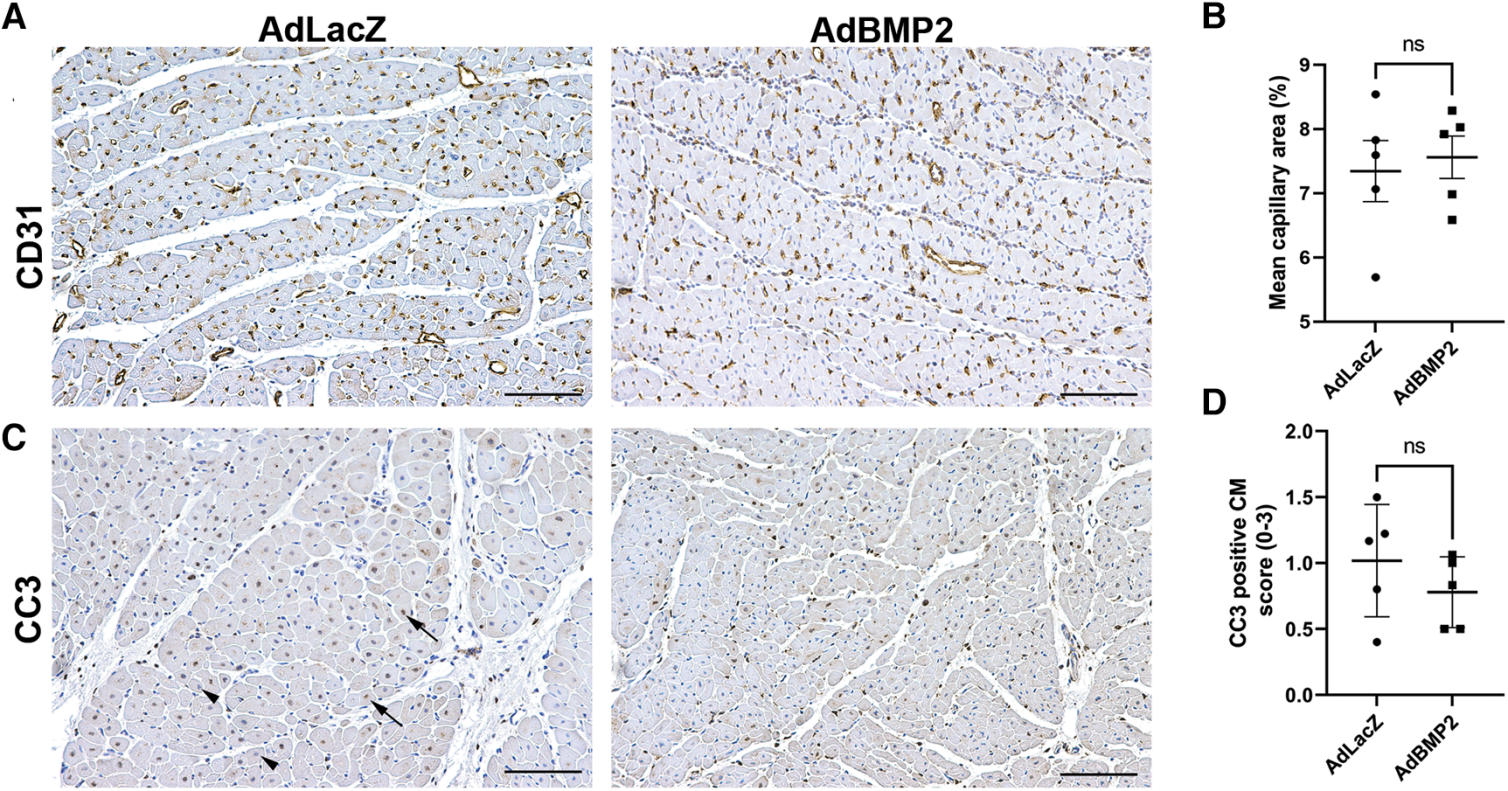
BMP2 does not induce angiogenesis or affect cardiomyocyte apoptosis in the ischemic pig myocardium. (**A**) Representative images of CD31-stained pig myocardium indicate that no changes in the amount or size of the capillaries were detected in the AdBMP2-treated ischemic pig myocardium in comparison to AdLacZ control samples in 6 days GT. Scale bars: 100 µm. (**B**) The mean capillary area was determined from CD31-stained ischemic myocardial thin sections at day 6 after GT (*n* = 5 pigs/group). No statistically significant difference was detected between the groups. The mean ± SEM values are shown. The Mann–Whitney *U* test was used to determine the statistical significance. (**C**) Representative images of CC3-stained myocardial samples at day 6 after AdBMP2 and AdLacZ GT (*n* = 5 pigs/group, AdLacZ = 30 samples, AdBMP2 = 16 samples). Scale bars, 100 µm. Arrows point to CC3-positive cardiomyocyte nuclei and arrow heads to CC3-negative cardiomyocyte nuclei. (**D**) The number of CC3 expressing apoptotic cardiomyocytes was scored from whole slide myocardial thin sections at d6 after GT. AdBMP2 did not alter the number of CC3-positive cardiomyocytes in comparison to AdLacZ (*n* = 5 pigs/group). Scores 0–3 were used, where 0 = absent, 1 = low, 2 = moderate, and 3 = high amount of CC3-positive cardiomyocytes. The mean ± SD values are shown. The Mann–Whitney *U* test was used to determine the statistical significance.

To evaluate the effect of hBMP2 GT on angiogenesis, the number of blood vessels was measured from the ischemic myocardial samples by immunohistochemistry for CD31 and whole slide imaging of the tissue section. No significant changes in the amount of blood vessels or CD31-positive areas were seen in hBMP2 transgene expressing areas in comparison to the AdLacZ control group at day 6 after GT (Figures 5A,B). To evaluate the effects of GT on cell apoptosis and proliferation, the myocardial samples were stained with apoptosis marker caspase 3 (CC3) and proliferation marker protein Ki-67 (Ki-67). No significant changes in the number of CC3-positive apoptotic CMs (Figure 5C) or Ki-67-positive proliferating CMs (Supplementary Figures S5A,B) were detected in the AdBMP2- vs. AdLacZ-treated ischemic pig myocardium.

### 2.5. BMP2 induces inflammation but does not affect fibrosis

**Figure 6:**
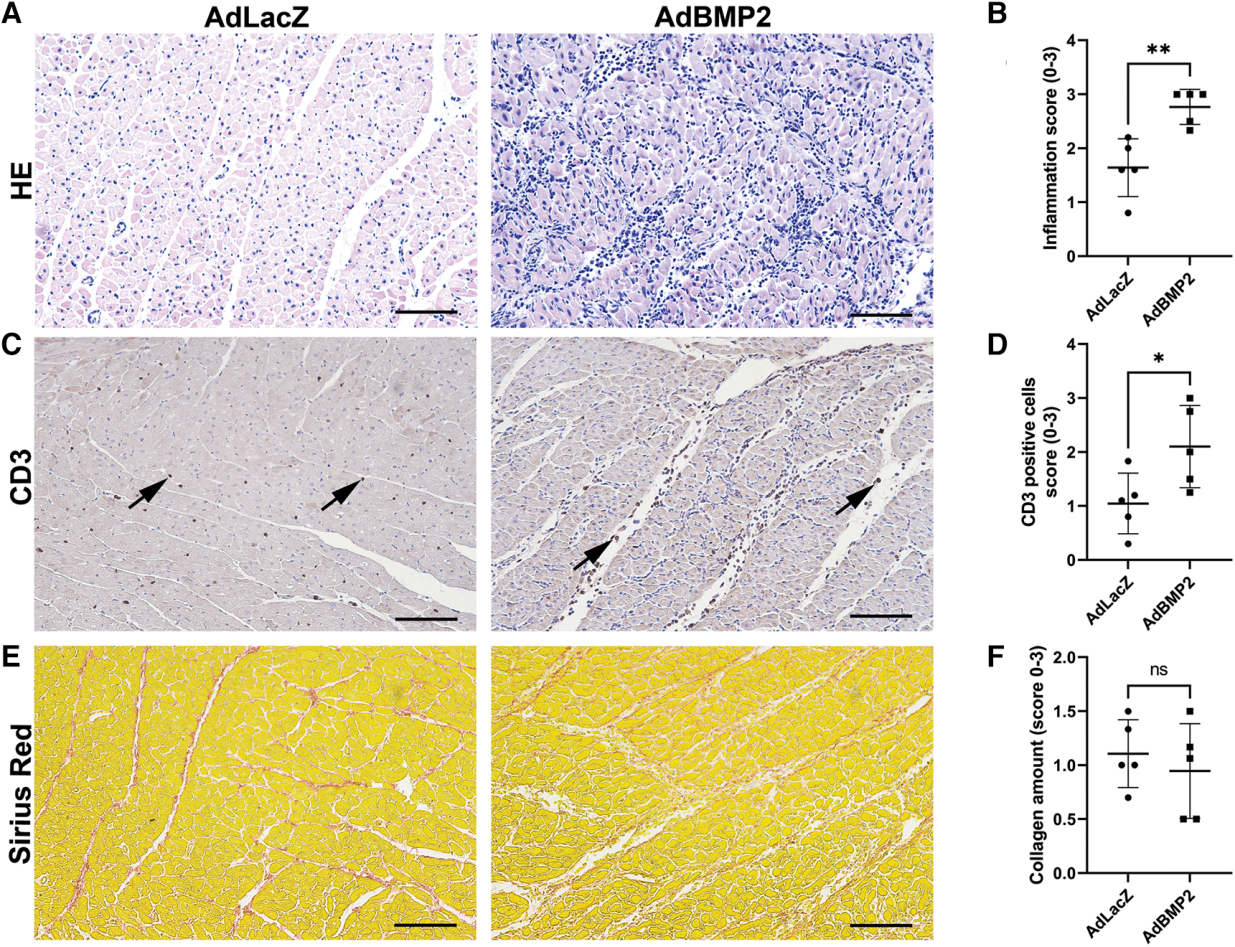
AdBMP2 GT induces inflammation but has no effect on fibrosis. (**A**) Representative images of H&E-stained ischemic pig myocardium treated with AdBMP2 or AdLacZ at day 6 after GT. AdBMP2 GT induced infiltration of inflammatory cells. Scale bars, 100 µm. (**B**) The level of inflammation after the GT at d6 was evaluated by scoring the amount of infiltrated inflammatory cells from H&E-stained ischemic myocardial whole slide samples. AdBMP2 GT significantly increased the inflammatory cell infiltration in comparison to AdLacZ GT (*n* = 5 pigs/group, AdLacZ = 23 samples, AdBMP2 = 16 samples). Scores 0–3 were used, where 0 = no cells, 1 = low, 2 = moderate, and 3 = high number of inflammatory cells. The mean ± SD values are shown. The Mann–Whitney *U* test was used to determine the statistical significance. **p < 0.01. (**C**) Representative images of CD3^+^ T cells in the ischemic myocardial samples at d6 after AdBMP2 and AdLacZ GT. AdBMP2 increased the amount of CD3^+^ cells (arrows) in the myocardium in comparison to AdLacZ. Scale bars, 100 µm. (**D**) T-cell infiltration scoring from CD3-stained myocardial tissue sections. AdBMP2 GT significantly increased the number of CD3^+^ T cells in comparison to AdLacZ (*n* = 5 pigs/group, AdLacZ = 23 samples, AdBMP2 = 16 samples). The mean ± SD values are shown. The Mann–Whitney *U* test was used to determine the statistical significance. *p < 0.05. (**E**) Representative images of Sirius red–stained myocardial samples after AdBMP2 or AdLacZ GT at day 6. Scale bars, 100 µm. (**F**) The level of fibrosis was evaluated by scoring the amount of collagen from AdBMP2- or AdLacZ-treated myocardial samples stained with Sirius red (*n* = 5 pigs/group, AdLacZ = 23 samples, AdBMP2 = 16 samples). AdBMP2 did not induce fibrosis. Scores 0–3 were used, where 0 = physiological amount of collagen, 1 = low increase of collagen, 2 = moderate increase of collagen, and 3 = high increase of collagen. The mean ± SD values are shown. The Mann–Whitney *U* test was used to determine the statistical significance.

To evaluate the degree of inflammatory response after AdBMP2 GT, myocardial samples were further quantitated from sections stained with H&E or CD3 antibody. AdBMP2 GT was shown to significantly increase the number of inflammatory cells in comparison to the AdLacZ control group (Figures 6A,B). Accordingly, more CD3^+^ T cells were detected in the AdBMP2 group and they were observed to localize between the CMs (Figures 6C,D). BMP2 transgene expression was detected in the same areas as inflammatory cells (Supplementary Figure S6). In addition, Ki-67 positivity was detected in cells resembling inflammatory cells, implying their expansion onsite (Supplementary Figure S5A). As BMP2 had been previously suggested to induce inflammation by regulating cell adhesion protein E-selectin via a TNF-α-mediated mechanism (37) and modulate VEGFR2 signaling (21) that could induce vascular permeability and thus explain the observed pericardial effusion after AdBMP2 GT, we determined TNF-α and VEGF-A mRNA expression by RT-qPCR from the GT areas. However, no statistically significant differences were detected between the GT groups (Supplementary Figures S7A,B). Finally, as inflammation could trigger fibrosis, collagen amount and TGFβ-1 mRNA expression, a known pro-fibrotic marker, were detected from the GT areas. No changes were detected between the AdBMP2 and AdLacZ groups in the amount of collagen detected by Sirius red staining (Figures 6E,F) or TGFβ-1 mRNA expression (Supplementary Figure S7C). Altogether, our data demonstrate that BMP2 GT causes inflammatory changes and pericardial effusion in ischemic myocardium, which does not support its therapeutic use in chronic CAD.

## 3. Materials and methods

### 3.1. Cells

Human umbilical cord vein endothelial cells (HUVECs) were isolated as previously described (38). The cells were cultured in an endothelial cell growth medium (Promocell, Heidelberg, Germany) on fibronectin–gelatin (10 µg/ml and 0.05%, respectively; Sigma-Aldrich, St. Louis, MO, USA)-coated cell culture dishes. Cells were used as passage <4 for the experiments. The Ethics Committee of Kuopio University Hospital approved the collection of umbilical cords for cell isolation (Kuopio, Finland, 341/2015).

### 3.2. Adenovirus vectors

AdBMP2, AdLacZ, and AdCMV vectors were generated and produced by the EATRIS-ERIC National Viral Vector Core Facility (NVVL, Kuopio, Finland). AdBMP2 and AdLacZ adenoviral vectors expressed their transgenes hBMP2 or beta-galactosidase (LacZ), respectively, under a CMV promoter. The AdCMV vector did not include a transgene and had only the CMV promoter. To confirm that the AdBMP2 vector is expressing and secreting its transgene, HUVECs were cultured on six-well plates (1.5 × 10^5^ cells/well) and transduced with 20 and 50 plaque-forming units of vector/cell (pfu/cell). Media samples were collected 24, 48, and 72 h post-transduction to measure the secreted hBMP2 protein using ELISA and WB.

### 3.3. Animal experiments

Three-month-old female domestic pigs were used. Ischemia (day −14) was induced by placing a bottleneck stent in the proximal part of the left anterior descending artery 14 days before the GT as previously described (34). To verify successful ischemia, we detected the blockage of LAD in every animal by angiography. ECG was monitored during all animal operations using a three-lead ECG and a patient monitor. To prevent ventricular arrhythmias, the animals received daily 200 mg of amiodarone (Cordarone®; Sanofi-Aventis, Helsinki, Finland) and 2.5 mg of bisoprolol (Bisoprolol ratiopharm; Ratiopharm GmbH, Ulm, Germany) starting 1 week before the ischemia operation until the end of the experiment. To prevent stent thrombosis, pigs were given a loading dose of clopidogrel (300 mg, Clopidogrel Mylan; Mylan S.A.S., Saint Priest, France) and acetylsalicylic acid (300 mg, ASA-Ratiopharm; Ratiopharm, Espoo, Finland) a day before the ischemia operation. During the operation, pigs received 100 mg of lidocaine (Lidocaine 10 mg/ml; Orion Pharma, Espoo, Finland) and 2.5 ml of MgSO_4_ (Addex-magnesium sulfate 246 mg/ml; Fresenius Kabi, Uppsala, Sweden) intravenously to prevent ventricular arrhythmias. Enoxaparin (30 mg) (Klexane®; Sanofi-Aventis) was given intravenously at the start of the ischemia operation to prevent blood coagulation and thrombosis. An intramuscular injection of 1.5 ml atropine (1 mg/ml; Leiras, Helsinki, Finland) and 6 ml of azaperone (Stresnil® 40 mg/ml; Janssen Pharmaceutica N.V., Beerse, Belgium) was used to sedate animals before the operations. Animals were put under general anesthesia using 15 mg/kg/h propofol (Propofol-Lipuro 20 mg/ml; B. Braun, Melsungen, Germany) and 10 µg/kg/h fentanyl (Fentanyl™ 50 µg/ml; Janssen-Cilag, Espoo, Finland) after the initial sedation. All animal experiments were approved by the Animal Experiment Board in Finland (ESAVI-2019-015164) and carried out in accordance with the guidelines of the Finnish Act on Animal Experimentation.

### 3.4. Gene transfer

Intramyocardial gene transfers (day 0) were performed 14 days after the ischemia operation (day −14), as described earlier (8). A NOGA MyoStar ® intramyocardial injection catheter (Biosense Webster, a Johnson & Johnson company, Diamond Bar, CA, USA) was introduced to the left ventricle via a femoral sheath under fluoroscopic guidance (GE Innova 3100IQ Pro, NY, USA). An electroanatomical map of the left ventricle was acquired using the NOGA® mapping system (Biologics Delivery Systems, a Johnson & Johnson company, Irwindale, CA, USA). Using this map as a guide, 1 × 10^12^ vp/heart of AdBMP2 or AdLacZ vectors (*n* = 5 pigs/group) was administrated in 10 injections (200 µl each) into hypokinetic but still viable areas of the left ventricle. Unipolar voltage over 5 mV was used as a criterion for viable myocardium, and the lowest available local linear shortening value, preferably <6% but minimally <12%, was used as a criterion for hypokinesia (39). Animals were randomly divided into the study groups after the ischemia operation.

### 3.5. Functional measurements

Strain analysis and measurement of pericardial fluid were performed with transthoracic echocardiography (EPIQ 7G, Philips, Netherlands) from the parasternal short-axis view of the heart. Images were acquired while the pig was lying on its left side. Strain analysis was performed before ischemia operation (day −14), GT (day 0), and sacrifice (day 6). The left ventricle strain values were measured at the levels of the mitral valve, papillary muscles, and apex using Automated Cardiac Motion Quantification (aCMQ) (EPIQ 7G, Philips). The average strain values measured from these three levels, i.e., global strain values, were used for the final analysis. The size of the pericardial effusion was measured at d6 from the mitral valve level in the end-diastole. The left ventricular EF was measured before ischemia, GT, and sacrifice. The angiogram workstation ventricle analysis program (Innova 3100^IQ^ 3D, GE Healthcare) was used to measure EF from the cine radiograms of the left ventricle filled with contrast medium. EF was measured under dobutamine-induced stress. The dobutamine (12.5 mg/ml; Hameln, Germany) infusion levels were increased until the target heart rate of 160 bpm was reached. The absolute change in EF or strain was calculated as the difference between values measured at the end (day 6) and the beginning (day 0) of the GT: absolute change = (value at day 6) − (value at day 0).

### 3.6. Sample collection

The pigs were sacrificed (day 6) using intravenous KCl under general propofol–fentanyl-induced anesthesia 6 days after the GT. Tissue samples from the heart were collected from the GT area and posterior wall. Safety samples were collected from the lung, liver, kidney, spleen, brain, retina, ovary, and thoracic and femoral plexus lymph nodes. Samples for histology were fixed in 4% paraformaldehyde (pH 7.2) for 48 h at 4°C and then placed in 15% sucrose for 48 h before embedding in paraffin. Samples for molecular biology measurements were snap-frozen in liquid nitrogen and stored at −70°C.

### 3.7. Validation of the GT area

The GT areas were collected using digital NOGA maps created during the vector injection. The GT areas were validated using IF staining with hBMP2 antibody. hBMP2 expression was scored qualitatively from the hBMP2 IF-stained myocardial samples. A scale from 0 to 3 was used to score BMP2 expressions, where 0 means no expression and 3 high expression (Supplementary Figure S8). Samples with scores 2 or 3 were used as validated AdBMP2 GT areas. Altogether, 16 samples from five AdBMP2-treated pigs and 23–30 samples from five AdLacZ-treated pigs were used in the analysis. No hBMP2 expression was detected in the AdLacZ samples.

### 3.8. Histology and immunohistochemistry

The Supplementary Material (Supplementary Table S1) lists the details of the used antibodies. Paraffin-embedded tissue samples were cut into 4 µm thin sections. Myocardial samples were stained with H&E, Sirius red, or antibodies for BMP2, CD31, CC3, CD3, and Ki-67. Stained sections were imaged using a Hamamatsu slide scanner (Biobank of Eastern Finland, Kuopio, Finland). Qualitative scoring of inflammation was performed from H&E- and CD3-stained samples, collagen amount from Sirius red–stained samples (fibrosis), and apoptotic cells from CC3-stained samples. The degree of inflammation or fibrosis and number of CD3^+^ T cells and CC3^+^ CMs were scored on a scale of 0–3 (0 = absent, 1 = low, 2 = moderate, and 3 = high) from the whole section area. The number of CD31-positive capillaries was analyzed using the Microvessel Algorithm in Aperio ImageScope (Leica, Wetzlar, Germany) from myocardial cross-sections. The analysis was performed from 16,725-µm^3^-sized image areas, with a total of 68 images from the AdBMP2 group and 151 images from the AdLacZ group. Ki-67-positive CMs were counted from 1,000 µm × 700 µm regions (eight regions/sample). Other Ki-67-positive cell types were not included in the analysis (Supplementary Figure S4). An average of Ki-67-positive CMs/region in each sample was used to analyze pig-specific average values.

The occurrence of morphological changes in safety samples was analyzed qualitatively from H&E-stained samples from the lung, liver, kidney, spleen, brain, retina, ovary, and thoracic and femoral plexus lymph nodes. To specifically uncover morphological changes caused by hBMP2 transgene, H&E staining was performed for previously hBMP2 IF-stained and imaged (Eclipse Ni-E Nikon microscope, Tokyo, Japan) myocardial sections with high transgene expression. H&E staining was performed according to the standard protocol after removing coverslips with xylene.

### 3.9. Gene expression analysis

The Supplementary Material (Supplementary Table S2) lists the primers used in the gene expression analysis. Snap-frozen tissue samples from the myocardium were homogenized using Tri-reagent (Sigma-Aldrich, Saint Louis, MO, USA) in a tissue homogenizer (Qiagen, Hilden, Germany). RNA was extracted using Tri-reagent according to the manufacturer’s instructions. cDNA synthesis was performed using random hexamers (Thermo Fisher Scientific, Waltham, MA, USA) and RevertAid Reverse transcriptase (Thermo Fisher Scientific). The following TaqMan primers were used (Thermo Fisher Scientific): pig endogenous BMP2, VEGF-A, TGFβ-1, TNF-α, and HPRT. The StepOnePlus Real-Time PCR System (Applied Biosystems, Foster City, CA, USA) was used to run RT-qPCR. Relative mRNA expression levels were analyzed using the delta-delta CT method, and HPRT was used as an endogenous control gene.

### 3.10. ELISA

Transgene expression and secretion were detected using the hBMP2 ELISA kit (Invitrogen, Waltham, MS, USA) from HUVEC media based on the manufacturer’s instructions. Total protein normalization was not performed because of the high fetal bovine serum content of media.

### 3.11. Western blot

AdBMP2 transgene expression and secretion were confirmed using WB from the HUVEC media from samples collected at 24, 48, and 72 h post-transduction. hBMP2 antibody (LSBio, Seattle, WA, USA) was used to detect the transgene expression (Supplementary Table S1). The iBind™ Flex Western Device (Invitrogen) was used according to the manufacturer’s instructions.

### 3.12. Clinical chemistry

The safety of AdBMP2 GT was evaluated in clinical chemistry evaluations from pig serum. Serum was collected from each animal at three time points, namely, day −14 (ischemia operation), day 0 (GT start) and day 6 (GT end). Sample collections were performed prior the operations. The measurements for ALP and ALAT for liver functionality, CREA for kidney functionality, LDH for changes in metabolism, CRP for general infections, and TPI for indications of MI were performed by MOVET Oy (Kuopio, Finland).

### 3.13. Statistics

GraphPad Prism 9.5.1 software (San Diego, CA, USA) was used for the statistical analyses. The Mann–Whitney *U* test (undistributed data) and one-way ANOVA test with Tukey’s *post-hoc* test (normally distributed data) were used. Outliers were detected from the data using the ROUT method (undistributed data) or Grubb’s test (normally distributed data). A *p*-value of <0.05 was used to define the statistical significance.

## 4. Discussion

Here, we evaluated the potential of BMP2 gene therapy for treating chronic myocardial ischemia in a large animal model. We used adenoviral vector-mediated intramyocardial delivery of BMP2 in pig myocardium 14 days post-ischemia operation. Our results indicate that BMP2 does not improve heart function after ischemia or promote beneficial outcomes, such as angiogenesis, nor cardioprotective features. On the contrary, AdBMP2 gene therapy induced an inflammatory response and pericardial effusion in the pig heart.

Several pro-angiogenic and regenerative growth factors have been tested to achieve therapeutic vascular growth or improved CM function in the ischemic myocardium. Recently, we showed that BMP2 has a synergistic effect with VEGF-A by increasing endothelial cell sprouting *in vitro* (21). Previously, BMP2 has been suggested to stimulate angiogenesis in neonate mouse hearts after MI and to limit CM apoptosis in adult mouse hearts after MI (20, 32). Our results contradict these studies, as no angiogenesis nor changes in CM apoptosis were seen in our study with AdBMP2 GT in the adult ischemic pig myocardium. These differences may derive from the differential model systems (neonatal vs. adult, mouse vs. pig), form of delivered BMP2 (protein vs. viral vector), delivery routes (intravenous vs. intramyocardial), time point of BMP2 delivery post-MI operation, and the used end time point (acute vs. chronic ischemia). For example, Ebelt et al. used intravenous delivery of BMP2 protein an hour after acute MI and followed the outcomes at 5 and 7 days post-MI. In addition, D’Amato et al. used adeno-associated viral vector-mediated gene delivery immediately after acute MI and detected outcomes at 7 and 14 days post-MI. In contrast, we used intramyocardial adenovirus vector delivery 14 days post-chronic MI and followed outcomes 20 days post-MI. Thus, acute MI and chronic MI may respond differently to BMP2 stimulus. In addition, the function of BMP2 may be different in neonates and adult hearts, warranting further studies.

The goal in treating CAD is to improve heart function that is impaired due to ischemia. Interestingly, even with limited CM apoptosis post-MI, no improvements in heart functionality as measured by EF were observed in the earlier mouse study with BMP2 protein (32). Compared with mouse hearts, pig hearts allow better transgene targeting to the ischemic tissue and translation of the functional effects to humans. Furthermore, the same instrumentation and similar dosing of gene medicine can be used for humans and pigs. As in this study, gene therapy vectors are typically injected into the ischemic areas with reduced blood flow. The NOGA mapping system allows electromechanical heart mapping, followed by targeted injection of the gene medicine to the ischemia-affected myocardium. However, despite targeted gene delivery and using large animal models, we did not see improvements in heart function, measured as EF and strain. Previously, we have demonstrated improved perfusion of the heart with the same animal model and similar GT set-up used in this study, but with adenovirus vectors expressing other growth factors than BMP2 (6, 8).

Although the expression of endogenous BMP2 has previously been identified to be upregulated after acute MI in mice, pigs, and humans, its possible role in disease pathogenesis or recovery processes of the ischemic myocardium has remained largely unknown (21, 37, 40). Furthermore, Sanders et al. showed that endogenous BMP2 is upregulated during the inflammatory phase after acute MI in mice and that BMP2 induces monocyte adhesion to endothelial cells *in vitro* (37). These results suggest that BMP2 regulates inflammation, but direct mechanical evidence is still lacking *in vivo*. Here, we show for the first time that BMP2 induces an inflammatory response in the ischemic myocardium. Inflammatory cells were specifically found in the areas with increased BMP2 transgene expression, supporting that BMP2 transgene expression induces inflammation. Since inflammation-induced fluid accumulation in the pericardium, i.e., pericardial effusion, can prevent the left ventricle from filling properly and lead to cardiac tamponade (41), the induction of pericardial effusion is a severe adverse effect of the AdBMP2 GT. The therapeutic use of BMP2 protein in bone regeneration has previously been associated with swelling and seroma formation, including life-threatening cervical spine swelling in patients (42, 43). Even though no defects in left ventricle functionality, blood clinical chemistry, nor safety tissue morphology were detected in this study, inflammation and pericardial effusion are striking adverse side effects of AdBMP2 GT.

Aside from gene and protein therapy, the potential of BMP2 in heart regeneration cell therapies for MI has been studied. For example, *in vitro* pre-incubation of c-Kit+ mesenchymal stem cells with BMP2 led to increased differentiation of functional CMs and improved heart functionality after cell transplantation in a rat MI model (33). Although not usable as an intramyocardial GT for treating myocardial ischemia due to induced inflammation and pericardial effusion, BMP2 may benefit cell therapies that induce cardiac regeneration after MI.

Taken together, our data demonstrate that BMP2 GT is unsuitable for inducing beneficial effects in adult myocardium after chronic ischemia and that BMP2 transgene induces pericardial effusion and inflammatory responses, which prevent its use in treating chronic CAD.

## Data Availability

The original contributions presented in the study are included in the article/**Supplementary Material**, further inquiries can be directed to the corresponding author.
