# Supplementary material for: BMP2 gene transfer induces pericardial effusion and inflammatory response in the ischemic porcine myocardium
Source: Front Cardiovasc Med. 2023 Nov 3;10:1279613. doi: 10.3389/fcvm.2023.1279613 (PMC10655027; doi:10.3389/fcvm.2023.1279613)
Supplement: Supplementary file 1 [file Table1.pdf]

Supplementary Material

1 Supplementary Figures

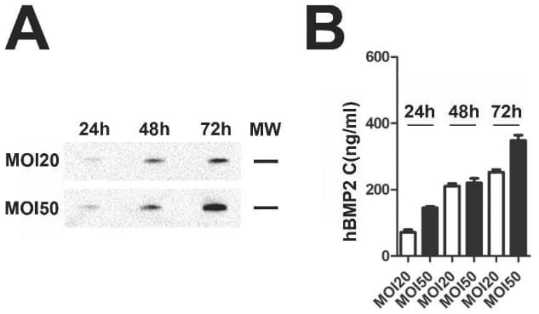

Supplementary Figure S1. BMP2 protein is secreted to medium after transducing cells with AdBMP2 vector. HUVECs were transduced with AdBMP2 and AdCMV vectors (20 or 50 MOI= pfu/cell) and the secretion of hBMP2 protein was detected from media collected 24, 48 and 72h post transduction. **A)** AdBMP2 transduction led to secretion of hBMP2 to media in all timepoints detected by WB (hBMP2 Ab). No hBMP2 was detected with AdCMV control vector. 15 kDa molecular weight marker band (MW) is shown **B)** Secreted hBMP2 protein in the media was also detected by ELISA. Mean ± SEM values are shown.

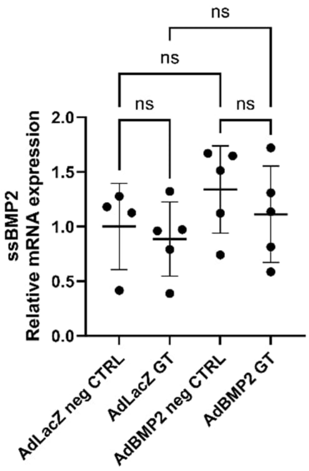

Supplementary Figure S2. Endogenous BMP2 mRNA expression does not change after chronic myocardial ischemia or AdBMP2 GT. Pig endogenous BMP2 mRNA expression (ssBMP2) was measured by RT-qPCR from AdLacZ and AdBMP2 treated pig myocardium at d6 after GT (n= 4-5 pigs/group, 20-21 myocardial samples per group) from GT area and non-GT area (neg CTRL; posterior wall of the left ventricle). No statistically significant differences were detected between GT groups or areas (GT area vs non-GT area). Mean ± SD values are shown. ANOVA test with Tukey's post-hoc test was used to determine the statistical significance.

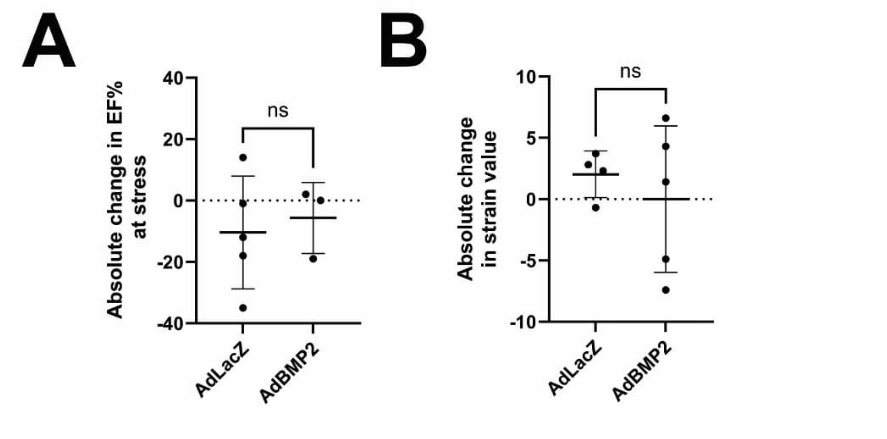

Supplementary Figure S3. AdBMP2 does not induce changes in left ventricle functionality. Absolute changes in left ventricle ejection fraction (EF) at stress and in strain values were determined as a change from d0 to d6. AdBMP2 GT did not alter the left ventricle functionality in comparison to AdLacZ GT measured as EF at stress (A) or with strain (B) (n=3-5 pigs/timepoint). Value for each pig (: dot) with mean ± SD values are shown. Mann-Whitney U test was used to determine the statistical significance.

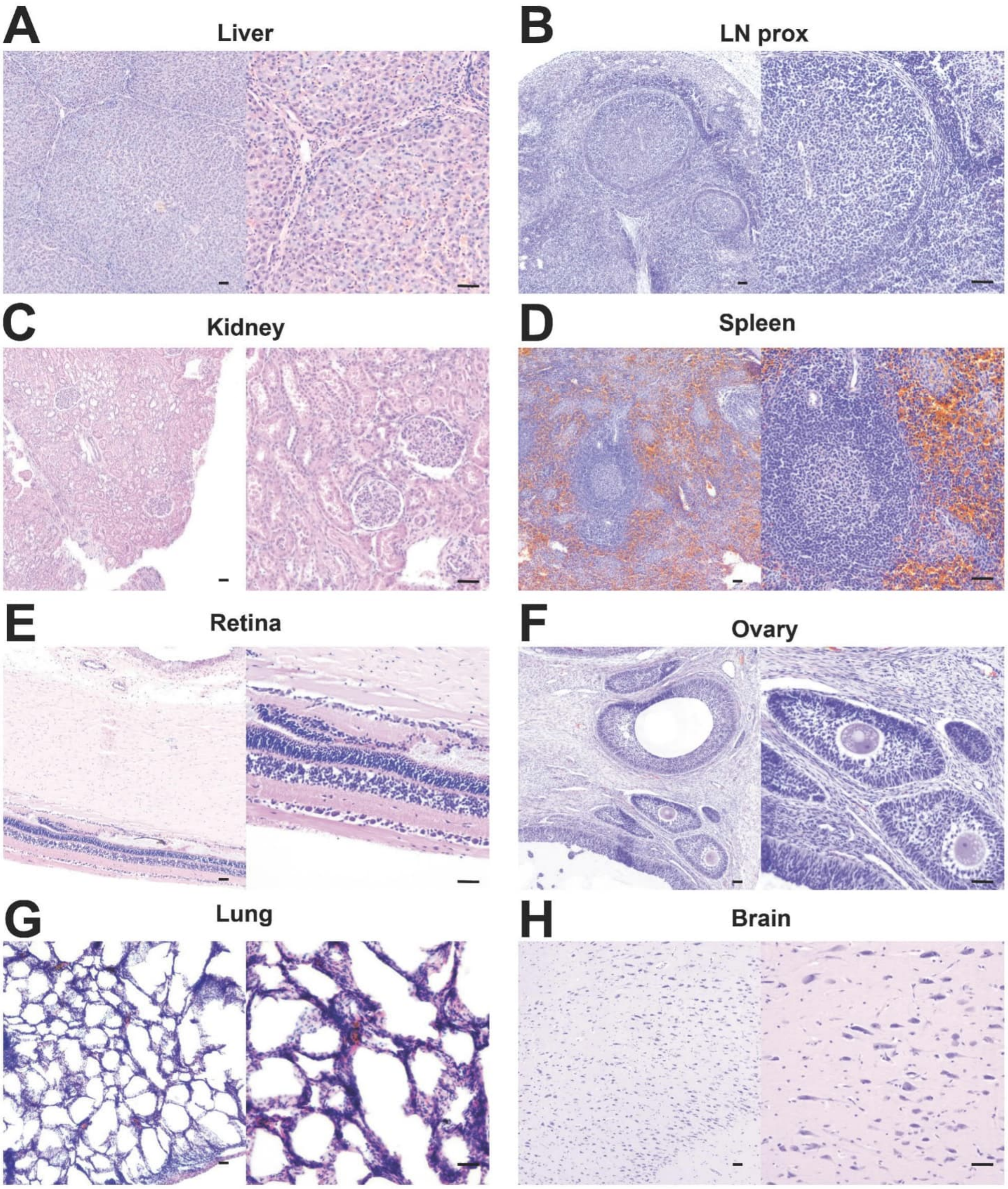

Supplementary Figure S4. Histology of selected safety tissues after AdBMP2 GT. H&E-stained tissues collected from AdBMP2 treated pigs at d6 after GT showing normal histology of the tissues. Liver (**A**), lymph node proximal (LN prox, **B**), kidney (**C**), spleen (**D**), retina (**E**), ovary (**F**), lung (**G**) and brain (**H**). Proximal lymph nodes were collected from the thoracic cavity. Scale bars, 100 μm.

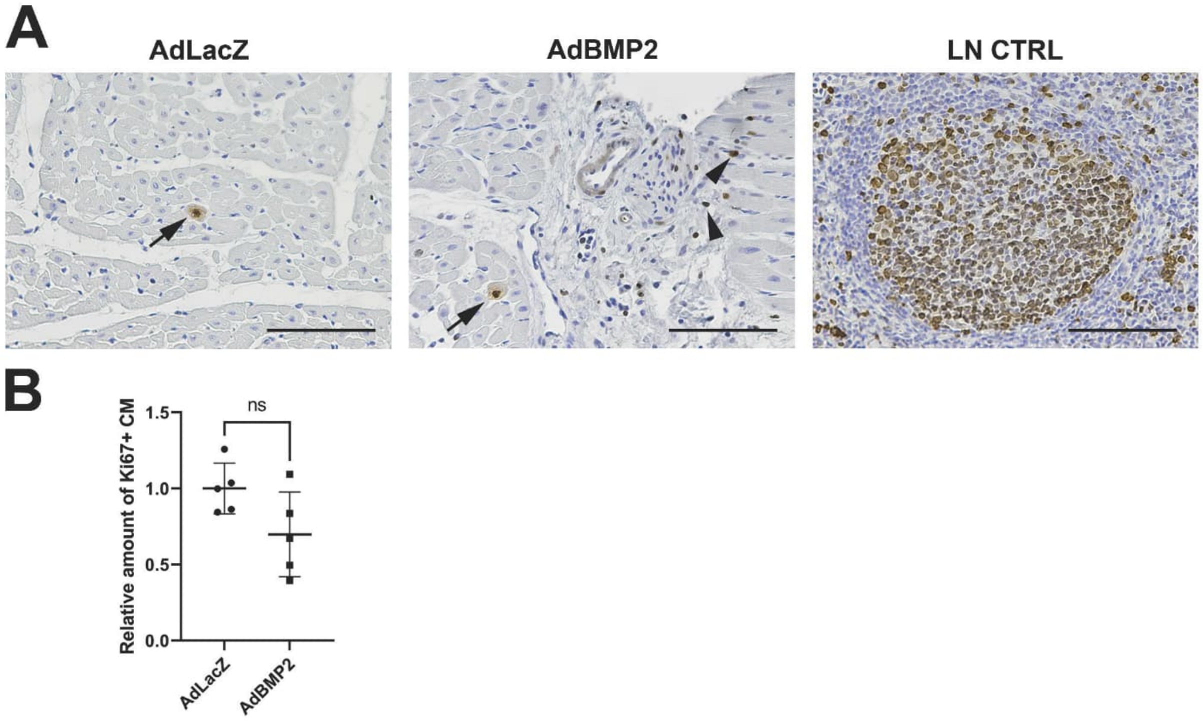

Supplementary Figure S5. BMP2 does not induce cardiomyocyte proliferation. A) Representative images of Ki-67-stained myocardial samples at d6 after AdBMP2 and AdLacZ GT. Ki67-positive cardiomyocytes (arrows) and lymphatic cells (arrowheads) were identified. Ki-67 staining of pig lymph node (LN CTRL) is presented on the right (positive control). Scale bars, 100 µm. **B)** Relative amount of Ki-67$^+$ cardiomyocytes was quantitated. No statistically significant difference was seen between AdBMP2 and AdLacZ groups (n=5 pigs/group, AdLacZ= 23 samples, AdBMP2= 16 samples). Mean ± SD values are shown. Mann Whitney U-test was used to determine the statistical significance.

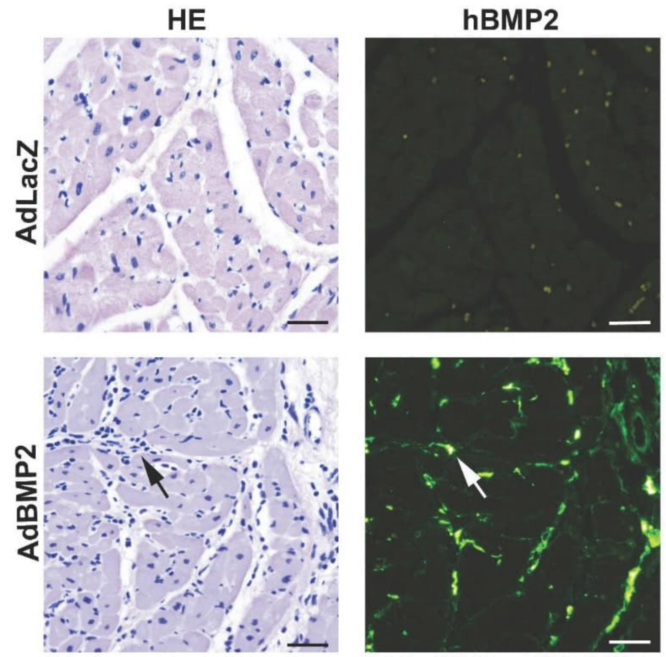

Supplementary Figure S6. hBMP2 protein expression locates to inflammation site. Representative images of H&E-stained and hBMP2 IF-stained (green) consecutive ischemic myocardial sections at d6 after AdBMP2 and AdLacZ GT. hBMP2 (white arrow, on the right) expression is seen in the same area as infiltrated immune cells (black arrow, on the left) in AdBMP2 treated myocardium. No expression of hBMP2 was seen after AdLacZ GT. Scale bars, 50 µm.

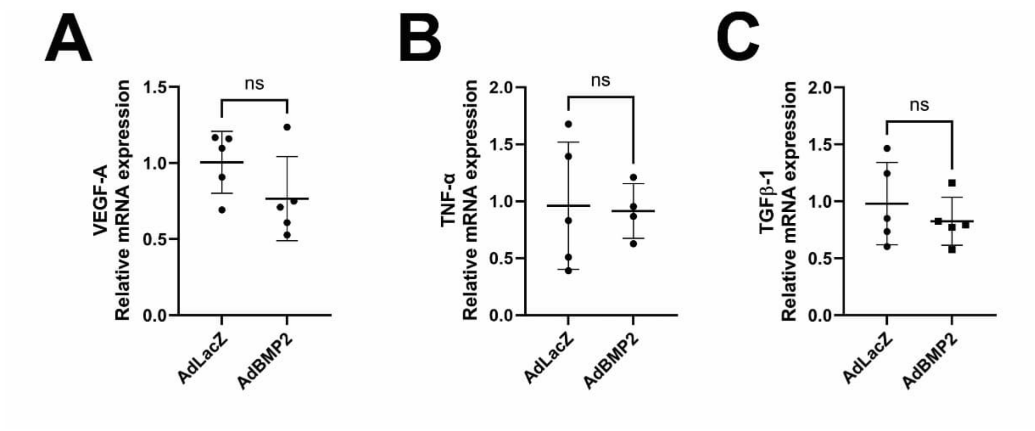

Supplementary Figure S7. Expression of endogenous VEGF-A, TNF-α and TGFβ-1 was unchanged after AdBMP2 GT. mRNA expression of endogenous VEGF-A (**A**), TNF-α (**B**), TGFβ-1 (**C**) were measured by RT-qPCR from AdLacZ and AdBMP2 treated pig myocardium at d6 after GT (5 pigs/group, 23-31 myocardial samples/pig). No statistically significant differences were detected between the GT groups. Mean ± SD values are shown. Mann-Whitney U test was used to determine the statistical significance.

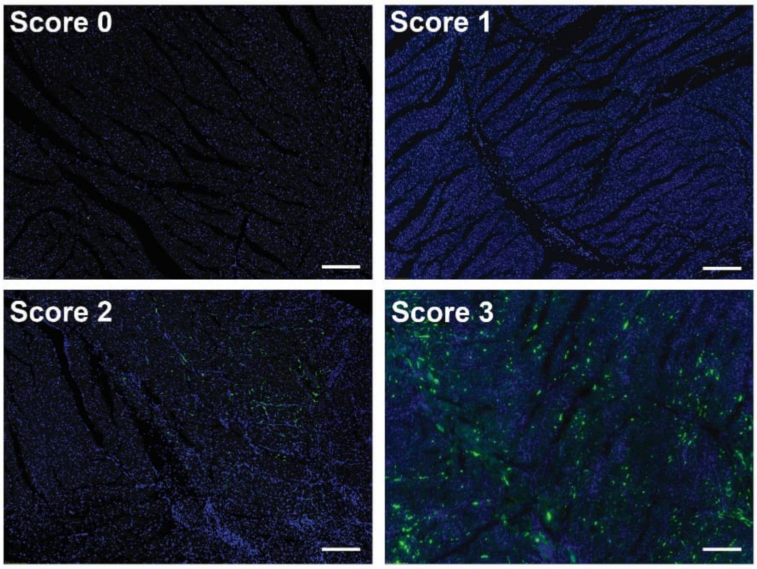

Supplementary Figure S8. Representative images of hBMP2 transgene expression levels in ischemic myocardium used for scoring. Transgene expression levels were scored from hBMP2 IF-stained (green= hBMP2 protein) myocardial thin sections with score values 0-3 (Score 0= no expression, Score 1= low expression, Score 2= moderate expression, Score 3= high expression). Blue color represents cell nuclei. Scale bars, 200µm.

2 Supplementary Materials & Methods

Supplementary Table S1. Antibodies used in IHC- and IF-stainings and WB.

Target antigen	Vendor or Source	Catalog #	Working concentration	Application
BMP2	LSBio	LS-C407899	1:250	IF
BMP2	LSBio	LS-C407899	1:1000	WB
CC3	Novus Biologicals	NB600-1235	1:200	IHC
CD3	Thermo Fisher Scientific	MA1-90582	1:200	IHC
CD31	Abcam	ab28364	1:50	IHC
Ki67	Abcam	ab16667	1:25	IHC

Supplementary Table S2. Primers used in gene expression analysis.

Gene	Full name	Assay ID	Vendor
ssBMP2	pig Bone morphogenetic protein 2	qSscCEP0042152	BioRad
ssHPRT	pig hypoxanthine phosphoribosyltransferase 1	Ss03388274_m1	Thermo Fisher Scientific
ssTGFβ-1	pig transforming growth factor beta 1	Ss04955543_m1	Thermo Fisher Scientific
ssTNF- α	pig tumor necrosis factor	qSscCIP0035447	BioRad
ssVEGF-A	pig vascular endothelial growth factor A	qSscCIP0026316	BioRad
